# Reduced probability of improving viro‐immunological state in subjects with vertical transmission of HIV reaching adult age: A multicenter retrospective cohort study

**DOI:** 10.1002/iid3.778

**Published:** 2023-02-09

**Authors:** Francesca Pennati, Stefano Calza, Antonio Di Biagio, Cristina Mussini, Stefano Rusconi, Stefano Bonora, Alberto Borghetti, Eugenia Quiros‐Roldan, Giovanni Sarteschi, Marianna Menozzi, Micol Ferrara, Anna Celotti, Arturo Ciccullo, Vania Giacomet, Ilaria Izzo, Laura Dotta, Raffaele Badolato, Francesco Castelli, Emanuele Focà

**Affiliations:** ^1^ Unit of Infectious and Tropical Diseases University of Brescia and ASST Spedali Civili Hospital Brescia Italy; ^2^ Unit of Biostatistics, Department of Molecular and Translational Medicine University of Brescia Brescia Italy; ^3^ Clinic of Infectious and Tropical Diseases University of Genova and “San Martino” Hospital Genoa Italy; ^4^ Department of Infectious Diseases University of Modena and Reggio Emilia and Modena Polyclinic Modena Italy; ^5^ Unit of Infectious Diseases University of Milano and ASST Fatebenefratelli “L. Sacco” Hospital Milan Italy; ^6^ Department of Infectious Diseases University of Torino and “Amedeo di Savoia” Hospital Turin Italy; ^7^ Infectious Diseases Unit Fondazione Policlinico Universitario Agostino Gemelli IRCCS Rome Italy; ^8^ Department of Safety and Bioethics, Section of Infectious Diseases Catholic University of the Sacred Heart Rome Italy; ^9^ Unit of Pediatrics University of Milano and ASST Fatebenefratelli “L. Sacco” Hospital Milan Italy; ^10^ Unit of Pediatrics University of Brescia and ASST Spedali Civili Hospital Brescia Italy

**Keywords:** antiretroviral therapy, discontinuation, HIV, vertical transmission, viro‐immunological effectiveness

## Abstract

**Introduction:**

Young adults with vertical transmission (VT) of human immunodeficiency virus (HIV) represent a fragile population. This study evaluates factors associated with viro‐immunological outcome of these patients.

**Methods:**

We performed a multicenter study including HIV‐infected subjects with VT ≥ 18 years old from six Italian clinics. Subjects were observed from birth to death, lost to follow‐up, or last visit until December 31, 2019. Condition of “optimal viro‐immunological status” (OS) was defined as the simultaneous presence of HIV ribonucleic acid (RNA) < 50 copies/mL, CD4+ > 500 cells/mm^3^, and CD4+/CD8+ ratio ≥ 1.

**Results:**

A total of 126 subjects were enrolled. At 18 years of age, 52/126 (44.4%) had HIV‐RNA > 50 copies/mL, 47/126 (38.2%) had CD4+ < 500/mm^3^, and 78/126 (67.2%) had CD4+/CD8+ < 1; 28 subjects (23.7%) presented in the condition of OS. Having a CD4+/CD8+ ratio ≥ 1 at 18 years of age was related with an increased probability of shift from suboptimal viro‐immunological status (SOS) to OS (HR: 7.7, 95% confidence interval [CI]: 4.23–14.04), and a reduced risk of shift from the OS to the SOS (HR: 0.49, 95% CI: 0.26–0.92). Acquired immunodeficiency syndrome (AIDS) diagnosis significantly reduced the probability of shift from a viro‐immunological SOS to OS (HR: 0.09, 95% CI: 0.03–0.30). Subjects who had not achieved an OS at 18 years of age had an increased risk of discontinuation of combination antiretroviral therapy (cART, *p* = .019).

**Conclusions:**

Only a small proportion of subjects with VT of HIV reached the adult age with “OS”. Transition to the adult care with a compromised viro‐immunological condition represents a negative driver for future optimal infection control, with a higher risk of discontinuation of cART and a reduced probability to improve the immunological status later in the years.

## Graphical abstract

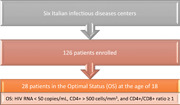

## 1 INTRODUCTION

The viro‐immunological effectiveness of combination antiretroviral therapy (cART) has drastically reduced perinatally infections in high‐income countries and has resulted in an increase in young adults with vertically transmitted human immunodeficiency virus (HIV). Today, vertical infection is a very rare event in high‐income countries and occurs in particular clinical situations. However, there is still a proportion of patients, especially in resource‐limited settings, who become infected through vertical transmission.^1^, ^2^, ^3^

Compared to peers with adult‐acquired HIV infection, adults with VT HIV represent a different population: more immunologically fragile, with an increased incidence of comorbidities, a lower adherence to antiretroviral therapy, a reduced development of intellectual abilities, and a tendency to underestimate the severity of the disease. Several studies showed how these individuals are characterized by a worse course of HIV infection, with a higher incidence of acquired immunodeficiency syndrome (AIDS) events, higher AIDS‐related mortality, as well as a higher incidence of comorbidities linked to the systemic effects of HIV infection along with a prolonged proinflammatory state, and long‐term toxicities of antiretrovirals.^4^, ^5^, ^6^, ^7^, ^8^, ^9^, ^10^

The main driver of AIDS‐related mortality and AIDS occurrence reduction are, on one hand, the effectiveness of antiretroviral therapy and, on the other hand, the achievement of a full immunological recovery with values of CD4+ T‐cell count > 500 cells/mm³ and a CD4+/CD8+ ratio ≥ 1.^11^, ^12^, ^13^, ^14^ These parameters, in fact, do not only indirectly express the state of immune system, but they are also surrogate markers of immune‐activation and chronic inflammation.^15^, ^16^, ^17^, ^18^ The importance of full immunological recovery in the VT HIV infection is still not fully studied.^18^, ^19^ New data could help to identify determinants associated with the survival of these patients and this would allow the design of optimized care pathways to ensure they grow in comfort and with a satisfactory standard of care.

The primary objective of this study was to analyze the long‐term viro‐immunologic efficacy of cART in patients with VT HIV infection. The analysis was done on patients aged ≥ 18 years on December 31, 2020 by (1) evaluating virological suppression during the follow‐up; (2) evaluating the immunologic status of subjects during the follow‐up, comparing subjects who achieve full viro‐immunologic recovery and those who fail to achieve an optimal infection control.

## 2 METHODS

### 2.1 Study design

Retrospective longitudinal multicenter observational study involving six infectious diseases centers devoted to HIV care in Italy.*

Demographic, clinical and viro‐immunological data, information about history of antiretroviral therapy, AIDS diagnosis, presence of co‐infections and comorbidities, and data about the virus resistance profile were collected during routine clinical practice. An anonymized electronic database has been created, including data available during follow‐up at Pediatrics (where subjects were in care until 18 years of age) and those during follow‐up at Adult Infectious diseases care unit. Study end‐points were evaluation of:

1) virological suppression, defined as HIV ribonucleic acid (RNA) < 50 copies/mL;
2) immunological performance, assessed by measurement of CD4+ T cell‐count divided into three categories: <200 CD4+/mm^3^, 200–500 CD4+/mm^3^, >500 CD4+/mm^3^, and of the CD4+/CD8+ ratio, defined as normal if ≥ 1 and not normal if < 1;
3) condition of “optimal viro‐immunological status” (OS), defined as the simultaneous presence of HIV RNA < 50 copies/mL, CD4+ T‐cell count > 500 cells/mm^3^, and CD4+/CD8+ ratio ≥ 1. Any other combination (HIV RNA ≥ 50 copies/mL and/or CD4+ T‐cell count ≤ 500 cells/mm^3^ and/or CD4+/CD8+ ratio < 1) as a suboptimal viro‐immunological status (SOS).

### 2.2 Inclusion/exclusion criteria

Inclusion criteria were (1) evidence of vertical transmission of HIV infection (evaluated considering the evidence of chronic HIV infection, confirmed by at least two molecular tests for HIV detection by the age of 2 years, and the HIV infection diagnosis in the mother) and (2) age ≥ 18 years. There were no exclusion criteria.

All subjects meeting the inclusion criteria and who had been referred to adult infectious diseases in the six centers considered were enrolled. Patients were observed from birth (or the earliest date of follow‐up available) until the date of death (if it occurred after 18 years of age), loss to follow‐up, last available follow‐up by December 31, 2019. Patients with >365 days of missing information who were not subsequently follow‐up were considered lost to follow‐up. The data collection was med between January 2018 and September 2019.

### 2.3 Statistical analysis

A preliminary descriptive analysis of the sample was performed using common indexes according to the type of variables: mean ± standard deviation, median and interquartile range for quantitative, percentages for qualitative variables. The OS event was modeled using a multistate Markovian model assuming that a subject could repeatedly transit over time from a stage of optimal infection control to a suboptimal one and vice‐versa. The therapeutic “switch” was modeled using a Cox model for recurrent events. Time‐dependent variables were associated with each time interval (between start and end of treatment regimen) considering the known value before the date of “switch” (discontinuation). Results are reported as hazard ratio (HR) and associated 95% confidence interval (CI). All analyses were two‐tailed at a 5% significance level.

### 2.4 Ethical issues

The present study was approved by Ethics Committee of Brescia and acknowledged by the Ethics Committees of the participating centers, in accordance with current regulations (Legislative Decree no. 211 of June 24, 2003 and subsequent additions and authorizations), conducted in full respect of human dignity and fundamental rights as dictated by the “Declaration of Helsinki,” by the standards of “Good Clinical Practice” issued by the European Community (as implemented by the Italian Government and in accordance with the Guidelines issued by the same bodies), in implementation of what is also provided for by the Council of Europe Convention for the Protection of Human Rights and Dignity of the Human Beings in the application of biology and medicine made in Oviedo on April 4, 1997.

Patient data have been made anonymous (alpha‐numeric code) in observance of the rights provided for by privacy legislation (Legislative Decree no. 196/2003 Art. 7). Since this study was retrospective and nonpharmacological, informed consent has not been provided because in Italy, ethical clearance for these studies is not needed (Italian Guidelines for classification and conduction of observational studies, established by the Italian Drug Agency, “Agenzia Italiana del Farmaco—AIFA” on March 20, 2008). In addition, we used the general authorization of the Italian Guarantor for the use of anonymized demographical and clinical data.

## 3 RESULTS

### 3.1 Population description

A total of 126 patients from the 6 participating centers were enrolled. Sociodemographic characteristics of the subjects are summarized in Table 1. Most of subjects (84.1%) were born before the introduction of cART in 1996. Of the 126 patients included in the study, 104 (82.5%) were in active follow‐up at the time of enrollment, 3 (2.4%) were deceased, and 19 (15.1%) were lost to follow‐up after 18 years of age. With regard to viro‐immunologic status, at the next closest date to the age of 18 years of age, 65/126 subjects (55.6%) had an HIV RNA value < 50 copies/mL, 76/126 (61.8%) had CD4+ T cells > 500 cells/mm^3^, 38/126 (32,8%) had a CD4+/CD8+ ratio ≥ 1. Overall, at the considered time point, only 28 subjects (23.7%) presented in the status of OS as previously defined.

**Table 1:** Sociodemographic characteristics of the sample sorted by sex.

	F (*n* = 70)	M (*n* = 56)	Total (*n* = 126)	*p* Value
Age, years				.361
Mean (SD)	28.19 (4.52)	27.43 (4.18)	27.85 (4.37)	
Range	18–37	19–36	18–37	
Nationality, *n* (%)				
Italy	59 (84.3%)	46 (82.1%)	105 (83.3%)	
Europe	3 (4.3%)	2 (3.6%)	5 (4.0%)	
Africa	5 (7.1%)	5 (8.9%)	10 (7.9%)	
Asia	1 (1.4%)	0 (0%)	1 (0.8%)	
Central‐South America	2 (2.9%)	3 (5.4%)	5 (4.0%)	
Status, *n* (%)				.577
Active follow‐up	57 (81.4%)	47 (83.9%)	104 (82.5%)	
Lost at follow‐up	12 (17.1%)	7 (12.5%)	19 (15.1%)	
Died	1 (1.4%)	2 (3.6%)	3 (2.4%)	
Drug addict, *n* (%)				.297
No	40 (57.1%)	37 (66.1%)	77 (61.1%)	
Yes	2 (2.9%)	2 (3.6%)	4 (3.2%)	
Ex	2 (2.9%)	4 (7.1%)	6 (4.8%)	
Unknown	26 (37.1%)	13 (23.2%)	39 (31.0%)	
Pregnancy, *n* (%)				
No	24 (34.3%)	NA	24 (19.0%)	
Yes	21 (30.0%)	NA	21 (16.7%)	
Unknown	25 (35.7%)	NA	25 (19.8%)	

Abbreviations: F, female; M, male; NA, not applicable; SD, standard deviation.

In total, 32/126 subjects (26.9%) were diagnosed with at least one AIDS‐related condition. The mean age at first diagnosis of an AIDS event was 14.22 years (±10), ranging from 0 to 30 years.

Regarding co‐infections, 3/119 (2.5%) subjects had hepatitis B virus (HBV)‐related chronic liver disease (HBsAg positive, HBV DNA detectable), of which 2 (1.7%) had vertical HBV acquisition; 17/85 (20%) had chronic hepatitis C virus (HCV) infection (HCV Ab positive, HCV RNA detectable), of which 13 had HCV maternal transmission; of these, 7 were exposed at least once to therapy with interferon, and 11 underwent eradicant therapy with direct‐acting antivirals (DAAs). Finally, 1/110 (0.9%) acquired syphilis in adulthood, and 1/121 (0.8%) was diagnosed with congenital syphilis.

In 65/126 (51.6%) patients, at least one comorbidity was present, and 7/126 (5.6%) had three comorbidities involving different districts simultaneously (Table 2).

**Table 2:** Distribution of comorbidities^a^ by district, in order of decreasing total frequency, broken down by sex.

District, *n* (%)	F (*n* = 70)	M (*n* = 56)	Total (*n* = 126)
Metabolic	10 (14.3%)	2 (3.6%)	12 (9.5%)
Bone	7 (10%)	5 (8.9%)	12 (9.5%)
Psychiatric/behavioral	3 (4.3%)	6 (10.7%)	9 (7.1%)
Cardiovascular	4 (5.7%)	5 (8.9%)	9 (7.1%)
Central/peripheral nervous system	3 (4.3%)	5 (8.9%)	8 (6.3%)
Kydney	1 (1.4%)	5 (8.9%)	6 (4.8%)
Other districts/patologies	24 (34.3%)	19 (33.9%)	43 (34.1%)

*Note*: Multiple measures per patient.

Abbreviations: F, female; M, male.

^a^ We defined a comorbidity as any relevant clinical event or disease affecting one of the organs or systems analyzed.

Considering the closest date to the age of 18, information about current antiretroviral therapy was available in 117/126 subjects, and in 69/117 information about previously taken therapies was available. Of these, 27/69 (39.1%) had been exposed to at least one non‐cART therapy regimen and 1/117 (0.9%) was still undergoing a non‐cART regimen at 18 years of age; 98/117 (83.8%) were taking a standard triple therapy regimen, 14/117 (12.0%) a regimen consisting of <3 molecules, and 5/117 (4.3%) a mega cART with >3 molecules on therapy. The ongoing regimens did not include a nucleoside/nucleotide reverse transcriptase inhibitor (NRTI, backbone‐free regimens) in 15/117 (12.8%) cases; a nonnucleoside reverse transcriptase inhibitor (NNRTI), a protease inhibitor (PI), an integrase strand transfer inhibitor (InSTI), or an entry inhibitor were included in the ongoing antiretroviral regimen in 43 (36.8%), 65 (55.6%), 30 (25.6%), and 2 (1.7%) cases, respectively.

The viral genotype for sequences involving reverse transcriptase inhibitors (NRTIs and NNRTIs) and PIs was available for 70 subjects. Among these, 31/70 (44.3%) had at least one major mutation for NRTIs, 24/70 (34.3%) had at least one for the NNRTIs, and 16/70 (22.9%) had at least one mutation for PIs. For 69 patients, the genotype for InSTI resistance was available, with only 1/69 (1.4%) having at least one mutation conferring resistance.

### 3.2 Viro‐immunological status

Based on the definition of OS and SOS, we assessed the probability of transition from one condition to another and the expected average time within each status.

The mean predicted residence time in the OS was 2.59 years (95% CI: 1.94–3.47), whereas that in the SOS was 6.91 years (95% CI: 5.29–9.02). The viro‐immunological status was evaluated at the age of 18. The probability for those in the suboptimal condition to remain in that status was 89% (95% CI: 85.8%–91%), whereas that of transit to the optimal status was 11% (95% CI: 8.8%–14%); for those who were in the OS, the probability of remaining in that status was 70% (95% CI: 62.6%–77%), whereas the probability of migrating to SOS was 30% (95% CI: 23.3%–37%).

These same parameters were assessed in the presence of some covariates. Sex has not been shown to have a significant impact on the risk of transit from both optimal to SOS and vice‐versa, with an HR of 0.86 (95% CI: 0.46–1.62) and 0.91 (95% CI: 0.52–1.59), respectively, as reported in Table 3.

**Table 3:** Expected mean residence time in suboptimal and optimal viro‐immunological states and probability of residence or transition from one status to another, divided by sex, CD4+/CD8+ ratio, HIV RNA, history of AIDS‐defining events, presence of resistance‐associated mutations.

	CD4/CD8 < 1 (*n* = 78)	CD4/CD8 ≥ 1 (*n* = 38)
Expected average time to remain in the SOS, years (SD)	9.35 (6.4–13.67)	1.21 (0.76–1.93)
Expected average time to remain in the optimal OS, years (SD)	1.29 (0.82–2.04)	2.66 (1.71–4.12)
Probability to remain in the SOS, % (95% CI)	92.9% (90.2%–94.9%)	52% (38%–64%)
Probability to remain in the OS, % (95% CI)	48.6% (32.8%–62.6%)	78% (71%–84%)
Probability of transition from SOS to OS, % (95% CI)	7.1% (5.1%–9.8%)	48% (36%–62%)
Probability of transition from OS to SOS, % (95% CI)	51.4% (37.4%–67.2%)	22% (16%–29%)

	HIV RNA < 50 (*n* = 65)	HIV RNA ≥ 50 (*n* = 52)
Expected average time to remain in the SOS, years (SD)	4.3 (3.0–6.15)	6.23 (3.45–11.25)
Expected average time to remain in the optimal OS, years (SD)	3.5 (2.42–5.08)	0.57 (0.32–1.03)
Probability to remain in the SOS, % (95% CI)	82% (75%–87%)	92.8% (89.9%–95%)
Probability to remain in the OS, % (95% CI)	78% (70%–84%)	21.9% (10.1%–42%)
Probability of transition from SOS to OS, % (95% CI)	18% (13%–25%)	7.2% (4.8%–10%)
Probability of transition from OS to SOS, % (95% CI)	22% (16%–30%)	78.1% (58.4%–90%)

	Absence of AIDS (*n* = 87)	Presence of AIDS (*n* = 32)
Expected average time to remain in the SOS, years (SD)	4.64 (3.51–6.12)	50.5 (16.19–157.48)
Expected average time to remain in the optimal OS, years (SD)	2.28 (1.67–3.11)	6.82 (2.2–21.13)
Probability to remain in the SOS, % (95% CI)	84% (80%–88%)	98.2% (95%–99%)
Probability to remain in the OS, % (95% CI)	68% (60%–75%)	86.5% (64.67%–95%)
Probability of transition from SOS to OS, % (95% CI)	16% (12%–20%)	1.8% (0.58%–5%)
Probability of transition from OS to SOS, % (95% CI)	32% (25%–40%)	13.5% (4.65%–35%)

	Absence of RAMs (*n* = 36)	Presence of RAMs (*n* = 34)
Expected average time to remain in the SOS, years (SD)	6.36 (4.72–8.67)	9.4 (5.21 –6.94)
Expected average time to remain in the optimal OS, years (SD)	2.38 (1.73–3.28)	3.72 (1.84–7.51)
Probability to remain in the SOS, % (95% CI)	88% (84.7%–91%)	91.1% (84.3%–95%)
Probability to remain in the OS, % (95% CI)	68% (59.5%–76%)	77.6% (61.3%–88%)
Probability of transition from SOS to OS, % (95% CI)	12% (9.1%–15%)	8.9% (5.1%–16%)
Probability of transition from OS to SOS, % (95% CI)	32% (24.3%–40%)	22.4% (12.3%–39%)

Abbreviations: AIDS, acquired immunodeficiency syndrome; CI, confidence interval; HIV, human immunodeficiency virus; OS, optimal viro‐immunological status; RAM, resistance‐associated mutation; RNA, ribonucleic acid; SOS, suboptimal viro‐immunological status.

Patients with a CD4+/CD8+ ratio ≥ 1 at 18 years of age had an increased probability of improve their viro‐immunological condition, transiting from SOS to OS (HR: 7.7, 95% CI: 4.23–14.04), and a reduced risk of shift from the OS to SOS (HR: 0.49, 95% CI: 0.26–0.92).

On the other hand, having an HIV RNA value ≥ 50 copies/mL at 18 years of age did not impact the likelihood of transit from SOS to OS (HR: 0.69, 95% CI: 0.35–1.38), but it increased the risk of shift from OS to SOS (HR: 6.13, 95% CI: 3.04–12.36).

The presence of a history of AIDS diagnosis significantly reduced the probability of transit from SOS to OS (HR: 0.09, 95% CI: 0.03–0.30), whereas it had no significant impact on the risk of migration from the OS to SOS (HR: 0.33, 95% CI: 0.10–1.08).

Finally, patients who had at least one major mutation conferring resistance to any class of antiretrovirals (resistance‐associated mutations, [RAMs]) had no significant impact on the probability of transit from one viro‐immunological condition to another (SOS to OS—HR: 0.68, 95% CI: 0.35–1.31; OS to SOS—HR: 0.64, 95% CI: 0.30–1.39).

The expected average time to remain in each status and the probability of maintaining or changing a viro‐immunological condition are shown in Table 3.

The presence of coinfections as well as the presence of comorbidities did not have a significant impact on the probability of either transit from the suboptimal condition to the optimal nor vice‐versa.

Assessing the risk of cART discontinuation, Cox model regression showed an increased risk of cART interruption or modification throughout all the observation period of the study in subjects who had not achieved OS (*p* = .019), who started from CD4+/CD8+ ratio < 1 (*p* = .003), who had a level CD4+ T cells ≤ 500 (*p* = .003) and an HIV RNA value ≥ 50 (*p* < .001), as shown in Table 4.

**Table 4:** Predictive factors for discontinuation or modification of any antiretroviral therapy regimen.

Variable	HR (95% CI)	*p* Value
Sex (M vs. F)	1.03 (0.82–1.29)	.835
AIDS (Yes vs. No)	1.31 (1.00–1.72)	.063
Presence of mutations (Yes vs. No)	1.10 (0.86–1.40)	.495
Optimal viro‐immunological status (Yes vs. No)	0.65 (0.49–0.88)	.019
CD4/CD8 ≥ 1 (Yes vs. No)	0.61 (0.46–0.81)	.003
CD4+ T lymphocyte ≥ 500 cells/mm³ (Yes vs. No)	0.66 (0.50–0.86)	.003
HIV RNA < 50 copies/mL (Yes vs. No)	0.52 (0.39–0.70)	<.001

Abbreviations: AIDS, acquired immunodeficiency syndrome; CI, confidence interval; F, female; HIV, human immunodeficiency virus; M, male; RNA, ribonucleic acid.

## 4 DISCUSSION

The results of this study showed that subjects with VT HIV infection tend to reach adulthood with a compromised viro‐immunological status, with a clear majority of subjects who have not reached an OS. We observed a high proportion of AIDS diagnosis already in pediatric and adolescent age, and a high proportion of subjects with comorbidities. Moreover, the study showed that subjects who did not reach OS at 18 years of age, had a low probability of achieving it later. The 15.4% of the sample was lost at follow‐up, similar or slightly higher than that described in HIV‐infected adult population.^20^, ^21^ This is in‐line with what is described in other studies about how the transition from pediatric to adult follow up represents a crucial moment from the point of view of retention in care for these subjects, with a deflection of adherence to follow‐up and therapy.^9^, ^22^, ^23^, ^24^

More than half of the enrolled subjects (51.6%) had at least one comorbidity and 5.6% had three comorbidities affecting different body districts. A South African study (a country in which the prevalence of VT HIV infection is significantly higher than in Italy) analyzed the prevalence of comorbidities compared to HIV‐negative adolescents and young adults of the same age.^6^ The study showed a prevalence of 43.5% of single comorbidity affecting a single district and 10.2% of multimorbidity. This aspect is particularly significant if we consider the young average age of the sample under examination, since these pathologies normally affect older subjects.

At the age of 18, all subjects for whom data were available were on cART, but only 44.4% had a confirmed HIV RNA < 50 copies/mL. This figure differs from the virological efficacy rate recorded in subjects with adult‐acquired HIV infection on therapy, which today in some settings reaches and exceeds 90%, and which in 2010 (the year closest to the mean age of 18 years in the sample studied) was described as ranging between approximately 60% and 70% in the industrialized countries.^25^, ^26^ This finding therefore highlights a likely lower adherence of these patients and a possible higher burden of drug resistance. A recent US study analyzed antiretroviral treatment adherence and the prevalence of unsuppressed HIV viremia^27^: the study showed that nonadherence increases significantly as we move from the preadolescent years to adulthood, from 31% to 50%, with a consequent increase in the prevalence of unsuppressed HIV viremia from 16% to 40%.

Regarding the achievement of the “optimal viro‐immunological condition,” this study has shown that only a low proportion (23.7%) of subjects with VT HIV infection reach that state at the age of 18. This finding is in‐line with the results of a recent Spanish study on a cohort of young people with VT HIV, whose immune system was found to have an increased activation and exhaustion profile, even after long‐term suppression under cART.^18^

From our data, we cannot say whether this result is due to the use, by subjects who had achieved OS, of more effective molecules or whether from being on therapy longer. What we do know is that overall in the patients included, although they were young, the average number of regimens of antiretroviral therapy taken was 5.6, with a maximum recorded number of 24 regimens; this finding is in‐line with that reported by a recent Italian study of the ARCA cohort on the prevalence of resistance‐associated genotypic mutations in adults with vertical HIV infection, which reported a mean number of regimens received of 5.^28^

In the dynamic modeling that analyzed the probability of transition from one state to another, we observed how the primary goal in the treatment of vertically acquired HIV infection is to immediately achieve full viro‐immunological recovery from the earliest stages of life and maintain it as long as possible.

The reported proportion of subjects with at least one resistance is high when compared with that of the general HIV‐positive population.^29^ A 2018 study analyzed a very large number (107,820) of genotypic sequences available in two of the largest public databases worldwide (Los Alamos and Stanford HIVdb), reporting a prevalence between 1996 and 2006 of resistance to NRTIs, NNRTIs, and PIs of 21%, 22%, and 12%, respectively, which is significantly lower than that found in our sample.^30^

This study has some strengths that it is important to emphasize: first of all, the sample size is considerable if we think of the low prevalence of vertical transmission of HIV in Italy. In addition, the follow‐up time of the study is very high. Many variables were considered, providing a very broad framing of the study subjects. Finally, the multicenter design limits the influence that the prescribing habits of a single center might have had on the management of the infection in the enrolled subjects.

However, the study has some limitations: the retrospective design limited the availability of clinical, viro‐immunological, and therapeutical information, especially related to the pediatric follow‐up years. The lack of this data may interfere with the evaluation of immunological damage in the study population. In addition, the lack of therapeutic characterization of the percentage of patients who achieved the OS endpoint does not allow us to dissertate on the reasons for that success: in particular, we cannot assess whether it is due to a long time on therapy or to the use of more effective molecules. Also, unfortunately, information on sociodemographic factors was very limited so the impact they might have on the history of these patients was not evaluated. The recorded proportion of deaths among the study population was low (2.4%); however, this finding certainly reflects the design of the study that aimed to observe precisely those subjects who survived to the pediatric age (the one with the highest mortality rates) and reached adulthood. Finally, the lack of comparison population, which is almost never available, except in subjects with documented seroconversion.

## 5 CONCLUSIONS

This study showed that reaching adult age with a compromised viro‐immunological status represents an obstacle to the subsequent achievement of an optimal one, despite the availability of a greater number of drugs with a better profile of both efficacy and tolerability. More efforts are needed to improve the adherence to cART of pediatric patients with HIV infection, and the retention in care of this particular group of people living with HIV.

## AUTHOR CONTRIBUTIONS

**Franesca Pennati**: Data curation; writing – original draft; writing – review and editing. **Stefano Calza**: Formal analysis; writing – review and editing. **Antonio Di Biagio**: Data curation; investigation; writing – review and editing. **Cristina Mussini**: Data curation; investigation; writing – review and editing. **Stefano Rusconi**: Data curation; investigation; writing – review and editing. **Stefano Bonora**: Data curation; investigation; writing – review and editing. **Alberto Borghetti**: Data curation; investigation; writing – review and editing. **Eugenia Quiros Roldan**: Data curation; investigation; writing – review and editing. **Giovanni Sarteschi**: Data curation; investigation; writing – review and editing. **Marianna Menozzi**: Data curation; investigation; writing – review and editing. **Micol Ferrara**: Data curation; investigation; writing – review and editing. **Anna Celotti**: Data curation; investigation; writing – review and editing. **Arturo Ciccullo**: Data curation; investigation; writing – review and editing. **Vania Giacomet**: Data curation; investigation; writing – review and editing. **Ilaria Izzo**: Data curation; investigation; writing – review and editing. **Laura Dotta**: Data curation; investigation; writing – review and editing. **Raffaele Badolato**: Data curation; investigation; writing – review and editing. **Francesco Castelli**: Data curation; investigation; writing – review and editing. **Emanuele Focà**: Conceptualization; supervision; writing – original draft; writing – review and editing.

## CONFLICT OF INTEREST STATEMENT

Emanuele Focà received speakers' honoraria, research grants, and advisory board fees from Viiv Healthcare, Janssen‐Cilag, Gilead Sciences, and Merck Sharp & Dohme. The remaining authors declare no conflict of interest.

## Data Availability

Data presented in the manuscript have not been published on the web. Data collected for this study are available for consultation on demand.

## Footnotes

* Unit of Infectious and Tropical Diseases, University of Brescia and ASST Spedali Civili Hospital, Brescia; Clinic of Infectious and Tropical Diseases, University of Genova and “San Martino” Hospital, Genoa; Department of Infectious Diseases, University of Modena and Reggio Emilia and Modena Polyclinic, Modena; Unit of Infectious Diseases, University of Milano and ASST Fatebenefratelli “L. Sacco” Hospital, Milan; Department of Infectious Diseases, University of Torino and “Amedeo di Savoia” Hospital, Turin;. Infectious Diseases Unit, Fondazione Policlinico Universitario Agostino Gemelli IRCCS Catholic, University of the Sacred Heart, Rome.
